# Supplementary material for: Increased expression of prokineticin 2 and its receptor in endometrium of recurrent implantation failure patients decreased the expression of MMP9 important for decidualization
Source: Reprod Biol Endocrinol. 2022 May 2;20:76. doi: 10.1186/s12958-022-00947-w (PMC9059424; doi:10.1186/s12958-022-00947-w)
Supplement: Supplementary file 1 — Additional file 1. Supplementary material-PCR. Original RT-qPCR data. [file 12958_2022_947_MOESM1_ESM.zip › 12958_947.docx]

Increased expression of prokineticin 2 and its receptor in endometrium of recurrent implantation failure patients decreased the expression of MMP9 important for decidualization

Jun Zhai^a*,b^ [bestzhai2005@163.com](mailto:bestzhai2005@163.com),
Linna Ma^a,b^ [15738398398@163.com](mailto:15738398398@163.com),
Ziyin Chang^a,b^ [2522042187@qq.com](mailto:2522042187@qq.com),
Ting Yu^a,b^ [yuting20211@163.com](mailto:yuting20211@163.com)

^a^Center for Reproductive Medicine
The First Affiliated Hospital of Zhengzhou University
Zhengzhou, China

^b^Henan Provincial Obstetrical and Gynecological Diseases (Reproductive Medicine) Clinical Research Center
The First Affiliated Hospital of Zhengzhou University
Zhengzhou, China

Abstract

Background

Studies have shown that abnormalities in the decidualization process were closely related to recurrent implantation failure (RIF). Prokineticin 2 (PK2) is a secreted protein with angiogenic and tissue remodeling functions but its role in the endometrium is unknown.

Methods

PK2 levels and its receptor PKR1 mRNA and protein levels in mid-secretory endometrium from normal and RIF women were examined by real-time PCR and western blotting, respectively. The effects of PK2 were evaluated by overexpressed PK2 in immortalized endometrial T-HESC cells using lentivirus vector and found different expression of Matrix metalloproteinase 9(MMP9) and lncRNA LUCAT1 by RNA-sequencing. The ability of PK2 to regulate LUCAT1 and MMP9 was verified in endometrial cells by real-time PCR and western blotting.

Results

Using endometrial biopsies from normal and RIF patients, we found increased expression of PK2, together with its receptor PKR1 in RIF patients. We then overexpressed PK2 in immortalized endometrial T-HESC cells using lentivirus vector and found decreased expression of Matrix metalloproteinase 9(MMP9), and increased expression of lncRNA LUCAT1. We verified the ability of PK2 to stimulate LUCAT1 and decrease MMP9 in endometrial cells. We further demonstrated that increased expression of a long noncoding RNA LUCAT1 and decreased expression of MMP9 in endometrial biopsies of patients with RIF. Thus, we highlighted the important role of PK2 and its receptor PKR1 in decidualization and RIF.

Conclusion

Prokineticin 2 and its receptor are important in endometrium decidualization. PK2 may affect endometrial decidualization through the LUCAT1- MMP9 pathway, thereby affecting embryo implantation.

Keywords

Prokineticin 2
Decidualization
LUCAT1
MMP9
Recurrent implantation failure

1. Introduction

In recent years, assisted reproductive technology has advanced, and the success rate per cycle has increased. However, 5 to 11% of patients remain infertile after multiple embryo transfers and recurrent implantation failure (RIF) has become a major obstacle to the further improvement of IVF outcomes [1]. The etiology of RIF is complex and involves abnormalities of gametes, embryos, uterus, immune system, and prothrombotic states [2, 3]. Moreover, the pathogenesis of most patients remains elusive.

Studies have shown that abnormalities in the decidualization process were closely related to RIF. Abnormal expression of cytokines and transcriptional regulators that regulate decidualization, such as Krüppel-like factor 12 (KLF12) [4], orphan nuclear receptor (Nur77) [5], C/EBPβ [6] and NOTCH signaling pathway [7] could lead to impaired embryo implantation and decidualization. Emerging evidence showed that the dysregulated expression of long non-coding RNA (lncRNA) is associated with RIF. Xu et al. found differential lncRNA expression between RIF and normal endometrium tissues, and showed that 169 lncRNAs were abnormally expressed in RIF., but the underlying mechanism was unclear.

Prokineticin 2 (PK2), a secretory protein with pleiotropic functions [8], acts on its G protein-coupled receptor to regulate various biological processes such as cell differentiation, angiogenesis, circadian rhythm, and sex hormone synthesis [9, 10]. PK2 could inhibit the transcription of adipokines and reduce the accumulation of adipose tissue [11]. The PK2 / PKR1 signaling pathway also promotes transformation of cardiac fibroblast progenitor cells into vasculogenic cells and maintains cardiac perfusion [12]. Prokineticin 2 has been detected in the endometrium, but its role in the endometrium remains unknown [13].

Long non-coding RNA (lncRNA) is a type of RNA molecule with a length of more than 200 nucleotides and participates in biological processes by interacting with macromolecules such as proteins to regulate gene imprinting, histone modification, cell cycle, RNA splicing and chromatin remodeling [14–16].

This study aims to explore the role of PK2 in the endometrium, to study whether PK2 affects endometrial decidualization by regulating lncRNA, to reveal the mechanism of embryo implantation failure from a new perspective of PK2/PK2 receptor and further explore potential therapeutic strategies.

1. Materials and method
   1. Sample collection

The study was approved by the Ethics Committee of the First Affiliated Hospital of Zhengzhou University (2019-KY-335) and all patients signed informed consent. From September 2018 to December 2018, patients under the age of 40 years treated at the Reproductive Medicine Center of the First Affiliated Hospital of Zhengzhou University were enrolled in this study. We recruited women with RIF (no clinical pregnancy after transfer of ≥4 good-quality embryos in ≥3 fresh or frozen cycles) and women with tubal factor who had at least one pregnancy history served as control group. All patients had normal ovarian reserve (FSH ≤ 10 mIU/ml, LH < 10 mIU/mL, E_2_ < 50 pg/mL on Day 3 of menstrual cycle), normal menstrual cycle (21 to 35 days). Women were excluded if they used contraceptive drugs or had intrauterine operation operations within 3 months. Also, those with endometriosis, uterine malformation, endometrial polyps, intrauterine adhesions, uterine fibroids, adenomyosis, hydrosalpinx, polycystic ovary syndrome (PCOS), immune diseases, prothrombotic, and endocrine diseases such as hyperprolactinemia were excluded. All patients were tested for serum sex hormones before transvaginal ultrasound to monitor follicle development and confirmed they were at day 7 of the luteal phase. Endometrial tissues were obtained 7 days after the LH using a Pipette endometrial suction curette. Endometrial tissues were washed repeatedly with normal saline immediately and each sample was divided into three sections for subsequent analysis.

- 1. Immunochemistry

Luteal phase endometrial tissue was embedded in paraffin. Tissue sections were placed in citric acid (PH6.0) for antigen retrieval and incubated with 3% H_2_O_2_ at room temperature in the dark for 25 min., washed three times with PBS for 5 min. Each before adding primary antibody (monoclonal rabbit anti-human PK2,PKR1,dilution of 1:200) (Abcam, America), overnight at 4C.Secondary antibody (HRT-labeled anti-rabbit IgG polymer) was incubated at room temperature for 50 min; sections were stained with DAB staining solution and counterstained with haematoxylin. Finally, sections were examined by microscopy.

- 1. Quantitative real-time PCR

Total RNA was extracted from endometrial tissue or T-HESC using TRIzol (Sigma, America) with total RNA content and concentration determined via spectrophotometry (Nano Drop 2000c). RNA was reverse-transcribed into cDNA by using a cDNA Reverse Transcription Kit (Takara Bio, Japan). Real-time PCR was carried out using SYBR Green (Takara Bio, Japan). Each sample was tested three times independently and each experiment was repeated three times. The relative expression level was analyzed using GAPDH as the internal reference gene. Primer sequences were listed in Table 1.

- 1. Western blotting

Frozen endometrial tissues or cells were lysed in 300ul lysis buffer containing a protease inhibitor (EpiZyme, Shanghai, China). After the protein concentration was determined by BSA method, SDS-PAGE (sodium dodecyl sulfate-polyacrylamide gel electrophoresis) was performed, and the protein was transferred to PVDF membrane. After TBST containing 5% fat-Free milk was blocked at room temperature for 2 h, the primary antibody was incubated, PKR1 antibody (Affinity, orb162427) 1: 500, PK2 antibody (Abcam, ab76747) 1: 200, MMP9 (N-terminal) Polyclonal antibody (Sanying,10,375-2-AP) 1: 100, IGFBP1 Polyclonal antibody (Sanying, 13,981-1-AP) 1:1000, PRL antibody (Abcam, ab188229) 1:2000, GAPDH Polyclonal antibody (Sanying, 10,494-1-AP) 1: 10000 overnight at 4 C. Next day, the PVDF membranes were washed by TBST 5-6 times* 5 min / per; were added horseradish peroxidase conjugated secondary antibody immunoglobulin G (IgG), were incubated at room temperature for 2 h, the membranes were finally revealed using electro-chemiluminescence (ECL) substrate and exposed to films.

- 1. Cell infection

Trypsin was used to digest T-HESC in logarithmic growth phase. T-HESC was adjusted to a density of 10^5^ / ml with DME / F-12 medium containing 10% FBS, and was inserted into a 25cm^2^ cell culture flask and incubated overnight at 37 °C. Lentivirus containing PK2 overexpressing vector was obtained from Hanheng Bio(Shanghai, China). T-HESCs were infected with the LV-PK2, or the control vector (NC). PK2-overexpressing T-HESCs were measured by RT-PCR.

- 1. SiRNA transfection

HESC was inoculated on the 6 - well plates, and the cells were cultured until the density reached about 75%. The transfection was completed by Lipofectamine RNAiMAX transfection Kit (Invitrogen). In brief, PK2- siRNA or scrambled siRNA was added to opti-MEM after vortex mixing and was kept at room temperature for 20 min. After 6 h, the 6 - well plate medium was replaced. After 48 h of transfection, PCR and Western-blotting were used to verify the siRNA transfection effect, which could be used for subsequent extraction of cell RNA and protein.

- 1. Induction of T-HESC decidualization in vitro

When the growth of cells reached about 90% confluency, cells were cultivated in DMEM / F12 medium for 48 h, and then were switched to fresh medium containing 8-Br-cAMP (0.5 mmol / l), MPA (1 μmol /l) to induce decidualization of the T-HESCs for 72 h. The inverted microscope was used to continuously observe the morphological changes of cells during decidualization.

- 1. RNA extraction and sequencing

According to the manual of RNeasy Mini Kit (250) Qiagen # 74106 kit, total RNA was extracted from decidualized cells. Agilent Bioanalyzer 2100 (Agilent technologies, Santa Clara, CA, US) was used for Quality Test. Magnetic beads containing Oligo (dT) was used to enrich the extracted mRNA, and rRNA (including cytoplasmic 28S, 18S, 5S rRNA and mitochondrial 12S, 5.8S rRNA) was removed from the total RNA, then RNA was used for non-coding RNA analysis. The remaining RNA included mRNA and lncRNA. After enriching and purifying mRNA, a cDNA library was constructed, and a eukaryotic strand-specific library, a lncRNA library, a SmallRNA library, etc. were also constructed. The library constructed by sequencing included mRNA and lncRNA data. Ensuring the temperature is appropriate, a breaking reagent was added to Thermomixer to break the mRNA into suitable fragments as templates to synthesize the first-strand cDNA, for the synthesis of the second-strand cDNA. The second-strand cDNA was purified and recovered with the sticky end repaired and the base “A” added to the 3 ‘end of the cDNA for linker is connection. After thisstep, the fragment size was selected, and finally PCR amplification was performed before Illumina NovaSeq 6000 sequencing.

- 1. Bioinformatics analysis

We used R software package under *P* ≤ 0.05, | log2FC | ≥1 to screen differential genes. GO and KEGG enrichment analysis were performed to reveal gene functions by using DAVID 6.8 tool (<https://david.ncifcrf.gov/>). STRING database was used to analyze the network analysis of protein- protein interactions.

- 1. Statistical analysis

The results were analyzed using SPSS version 22.0 or R software package version 3.2.2. The measurement data were represented as “mean ± SD”, and the t tests were performed for comparisons of two groups. The categorical data were performed using the χ2 test and Fisher’s exact test. Differences of *p* < 0.05 were considered statistically significant.

1. Results
   1. Expression of PK2, PKR1, PRL and IGFBP1 in endometrium

We used RT-qPCR and Western blotting to detect the expression of PK2, PKR1, PRL, and IGFBP1 in the luteal phase endometrium between the control group (*n* = 10) and the RIF group(n = 10). As compared with the control group, the decidualization markers PRL、IGFBP1 mRNA and protein levels were significantly reduced in RIF group: however, PK2 and PKR1 mRNA and protein expression levels were significantly increased (Fig. 1).

- - 1. Localization of PK2 and PKR1 protein in endometrial tissues

We used immunohistochemical staining to detect the location and expression of PK2 and PKR1 proteins in the two groups. The results showed that the PK2 and PKR1 were mainly located in glandular epithelial cells and stromal cells, and the expression of PK2 and PKR1 in the RIF group was significantly higher than that of the control group (Fig. 2).

- - 1. Effect of PK2 overexpression on the in vitro decidualization of T-HESC

To clarify the effect of PK2 on endometrial stromal cell decidualization, T-HESC was transfected with PK2-expressing lentivirus and control lentivirus. Forty-eight hours after transfection, they were cultured in 2% DCC-FBS-Free DEME /F12 medium containing 8-Br-cAMP (0.5 mmol/l) and MPA (SPELL OUT) (1 μmol/l) for 3 days. We next monitored morphological changes of the two groups at 0 h, 24 h, 48 h, and 72 h under inverted microscope. We found that the morphology of cells in the PK2 overexpression group was fusiform, and did not change significantly. In the control group, T-HESC gradually became larger and rounder from the spindle shape, nuclei increased, and the cell boundaries were blurred (Fig. 3A). Seventy-two hours after decidualization in vitro, we used RT-qPCR to detect the expression of decidualization markers PRL and IGFBP1. The expression levels of PRL and IGFBP1 mRNA in the PK2 overexpression group were lower than those of the control group (Fig. 3B).

- - 1. Identification of differentially expressed mRNAs and lncRNAs in PK2 overexpression group and control group

At 72 h, three decidualized endometrial stromal cell samples each were selected randomly from PK2-overexpression group and Control group for subsequent RNA-sequencing. A total of 266 mRNAs, 229 lncRNAs were detected with the criterion of |log2FC| > =1 and *p*-Value<=0.05. Among them,166 mRNAs, 194 lncRNAs, were significantly up-regulated, whereas 100 mRNAs, 35 lncRNAs were significantly down-regulated in the PK2-overexpression group compared with those in the control group. The Heatmap revealed that the PK2-overexpression samples were significantly different from the control samples based on the lncRNA data (Fig. 4A). The top 50 lncRNAs with the largest difference between the two groups and the arrow indicates LUCAT1 (Fig. 4B). Red area represents increased expression levels, and green represents decreased expression levels. Subsequently, we randomly selected 5 lncRNA (LINC02454, FMR1-AS1, LUCAT1, LINC01561, LINC00957) in the two groups for RT-qPCR verification. The results were consistent with RNA-seq analyses, confirming that the sequencing results were reliable (Fig. 4C).

- 1. PPI network construction

We imported the differentially expressed genes into the STRING website for PPI analysis. Among the mRNAs related to the decidualization process found in the PPI network included Matrix metalloproteinase 9(MMP9) and MMP9 can regulate the decidualization marker, IGFBP1 (Fig. 5).

- - 1. lncRNA-mRNA co-expression network analysis

The lncRNA-mRNA gene co-expression network was constructed based on the correlation analysis (Pearson correlation coefficient＞0.99, *P*＜0.05) between the differentially expressed lncRNA and mRNA. We found 82 lncRNAs were highly co-expressed with 90 mRNAs. According to the threshold of Pearson correlation coefficient＞ 0.7, *P* value < 0.1, we filtered out lncRNAs-MMP9 network involving 52 lncRNAs. Previous studies have found that LUCAT1 and RAMP2-AS1 are mainly involved in regulating cell cycle, cell proliferation and migration, especially LUCAT1. Many studies have confirmed that LUCAT1 is involved in regulating cell proliferation in various tissues. LUCAT1 and RAMP2-AS1 may affect stromal cell decidualization by regulating MMP9.

The expression levels of LUCAT1, RAMP2 - AS1 and MMP9 in the endometrium of the two groups were detected by RT-qPCR and Western blot. The results showed that compared with the control group, the LUCAT1 in the endometrium of the RIF group was significantly increased, the mRNA and protein levels of MMP9 were significantly decreased, and RAMP2 - AS1 had no significant change, indicating that the abnormal expression of LUCAT1 and MMP9 may play an important role in mediating the decidualization failure of RIF patients (Fig. 6).

- - 1. Effects of lentivirus overexpression of PK2 and PK2 - siRNA on the expression levels of LUCAT1 and MMP9

To investigate effects of over- and under-expression of PK2 on T-HESCs. Through the construction of empty vector + no decidualization group, empty vector + decidualization group, overexpression of PK2 + decidualization group, PK2-siRNA + decidualization group lentiviral transfection cell model in decidualization D6 days,RT-qPCR and Western blot were used to study the PK2 overexpression or knockdown of LUCAT1, MMP9 expression levels, the results showed that compared with empty vector decidualization group, PK2 overexpression group LUCAT1 RNA expression levels increased, MMP9 RNA and protein expression levels decreased, PK2-siRNA decidualization group LUCAT1 RNA expression levels decreased, MMP9 RNA and protein expression levels increased, further verify the PK2 regulation of downstream genes LUCAT1, MMP9 (Fig. 7).

1. Discussion

Endometrial decidualization is characterized by the transformation of stromal cells into large round or polygonal decidual cells [17] and impaired endometrial decidualization is closely related to infertility, implantation failure, and miscarriage [18–20]. PK2 has been demonstrated to be involved in multiple physiological and pathological processes. The PK2-PKR signaling system is expressed in the heart, kidney, uterus and other tissues, but its role in the endometrium is unclear [21, 22].

Battersby et al. showed that the expression of PK2 and PKR1 mRNA in the human endometrium did not change during the menstrual cycle, but the function of PK2 in endometrial tissue has not been studied [13]. Our results showed that the expression levels of decidualization markers PRL and IGFBP1 in endometrium of RIF patients in the middle luteal phase (7 days after LH peak) were significantly lower than those in control group, confirming that endometrial decidualization was defective in patients with RIF. Compared with the control group, the expression levels of PK2 and PKR1 were significantly elevated, and immunohistochemical results showed that higher staining of PK2 and PKR1 was detected in stromal cells than that in glandular epithelial cells, indicating that the abnormal expression of PK2 in endometrial stromal cells of RIF patients may be related to embryo implantation failure. To clarify the role of PK2 in the process of endometrial decidualization, we observed that changes in cell morphology were abnormal in the PK2-overexpression group after undergoing in vitro decidualization and the expression of PRL and IGFBP1 were dramatically reduced. Increased PK2 expression might lead to defective endometrial decidualization.

LncRNAs play important roles in a variety of biological processes, including epigenetics, transcription, post-transcription, and translation. At present, the study of the role of lncRNA in the decidualization process is in its infancy. Liang et al. have found that LINC473 could participate in endometrial decidualization. After knocking out LINC473, decidualization was impaired, suggesting that lncRNAs are involved in stromal cells decidualization [23]. The expression of LncRNAHand2os1 in mouse decidual cells at 6-8 days of pregnancy is increased and promotes the process of decidualization [24]. In placental trophoblasts of patients with recurrent miscarriage, the expression of Tristetraprolin (TPP) was also up-regulated, and TPP could inhibit the expression of lncRNA HOTAIR. HOTAIR was a regulatory factor regulating the proliferation, migration and invasion of trophoblasts, and the abnormality of HOTAIR leads to miscarriage [25].

In this study, we compared the expression level of lncRNA and mRNAs in T-HESC transfected with PK2-expressing lentivirus or negative control lentivirus by RNA-seq. We identified 229 lncRNA transcripts and 266 mRNA transcripts that were significantly changed in PK2-overexpression T-HESC group compared with those in the control group. GO and KEGG analysis found that differential mRNAs are mainly involved in phagocytosis, immune response regulating cell surface receptors, neutrophils mediated immune, and signaling pathways such as cell adhesion molecules, chemokines, and transendothelial migration of leukocytes. Using protein-protein interaction analysis of mRNA related to the decidualization, we also observed MMP9 constitutes the main network and MMP9 can regulate the decidualization-related gene IGFBP1.Matrix metalloproteinases (MMPs) are a family of proteases that are capable of degrading all major components of extracellular matrix [26]. MMPs secreted by endometrial stromal cells and decidual cells can degrade extracellular matrix and activate growth factors, thereby promoting the initiation of menstruation, regeneration and remodeling of the decidua [27].

We further found that LUCAT1was negatively correlated with MMP9. It has been shown that lncRNA is involved in regulating the proliferation, migration and apoptosis of cancer cells. LUCAT1 was highly expressed in breast cancer tissues, knockdown of LUCAT1 inhibited cell proliferation [28–30]. It is found that LUCAT1 is mainly involved in the regulation of cell cycle, cell proliferation, migration, while endometrial stromal cells undergo proliferation and differentiation during decidualization and require fine regulation of multiple cell factors.

It has been reported that LUCAT1 regulates MMP9 in Laryngeal squamous cell carcinoma cell lines, but its role in endometrial stromal cells remains to be studied [31]. There has been a lot of evidence that MMP9 plays an important role in the process of decidualization. Targeted knockout of MMP9 gene in mice can lead to a certain degree of impairment of endometrial decidualization and a reduction in the number of embryo implantations [27, 32]. Injecting an MMP9 inhibitor into the mouse uterine cavity prior to embryo implantation resulted in a slowdown in the development and remodeling of the mouse decidual region [33]. Therefore, we speculate that PK2 may regulate MMP9 through LUCAT1 to affect the decidualization.

We further study found that LUCAT1 was significantly higher and MMP9 was significantly lower in RIF patients than in normal endometrium. The abnormal expression of LUCAT1 and MMP9 may be the key factor in the failure of decidualization of RIF. In order to verify the regulatory effect of PK2 on LUCAT1 and MMP9, we found that the expression of LUCAT1 in the PK2 overexpression group was increased and the MMP9 level was decreased in the PK2 overexpression group and PK2 knock-down group. PK2-siRNA decidualization group showed the opposite trend. These studies suggested that the elevated PK2 in RIF patients may inhibit the expression of MMP9 through LUCAT1 to hinder the remodeling of extracellular matrix and thus the decidualization process, leading to the failure of embryo implantation. However, further experiments are needed to further explore the mutual regulatory mechanism of PK2, LUCAT1 and MMP9.

Acknowledgements

The authors particularly appreciate the help of the women who participated in this study. Thanks to Aaron J. W. Hsueh for his help in experimental designing and revising the articles, and thanks to Wenrong Dai and Yurong Cao for their assistance in the experiments.

Authors’ contributions

JZ, LM and ZC conceived and designed this study. LM, ZC and TY recruited the patients and collected endometrial samples. LM, ZC and TY performed the experiments. JZ, LM, ZC and TY analyzed the data. JZ, LM, ZC and TY supervised the research. LM and TY wrote the original draft. LM and TY edited the manuscript. All authors read and approved the final version of the manuscript.

Funding

This work was supported by the National Natural Science Foundation of China (Grant No. 82071649) and the Key Scientific Research Projects of Higher Education Institutions in Henan Province (Grant No.22A320025).

Availability of data and materials

The datasets that were used and/or analysed during the current study are available from the corresponding author upon reasonable request.

Declarations

Ethics approval and consent to participate

This study involving the use of human tissue specimens was approved by the Review Board of the First Affiliated Hospital of Zhengzhou University. The patient samples used in this study were obtained with informed consent.

Consent for publication

Not applicable.

Competing interests

Not applicable.

Conflict interests

The author(s) declared no potential conflicts of interest.

1. Additional file

Additional file 1 Supplementary material-PCR. Original RT-qPCR data.

References

1. Mak J, Chung C, Chung J, Kong G, Saravelos S, Cheung L, et al. The effect of endometrial scratch on natural-cycle cryopreserved embryo transfer outcomes: a randomized controlled study. Reprod BioMed Online. 2017;35(1):28–36.

2. Bellver J, Simón C. Implantation failure of endometrial origin: what is new? Curr Opin Obstet Gynecol. 2018;30(4):229–36.

3. Bashiri A, Halper K, Orvieto R. Recurrent implantation failure-update overview on etiology, diagnosis, treatment and future directions. Reprod Biol Endocrinol. 2018;16(1):121.

4. Zhang H, Zhu X, Chen J, Jiang Y, Zhang Q, Kong C, et al. Krüppel-like factor 12 is a novel negative regulator of forkhead box O1 expression: a potential role in impaired decidualization. Reprod Biol Endocrinol. 2015;13:80.

5. Huang C, Jiang Y, Zhou J, Yan Q, Jiang R, Cheng X, et al. Increased Krüppel-like factor 12 in recurrent implantation failure impairs endometrial decidualization by repressing Nur77 expression. Reprod Biol Endocrinol 2017;15(1):25.

6. Tamura I, Jozaki K, Sato S, Shirafuta Y, Shinagawa M, Maekawa R, et al. The distal upstream region of insulin-like growth factor-binding protein-1 enhances its expression in endometrial stromal cells during decidualization. J Biol Chem. 2018;293(14):5270–80.

7. Amjadi F, Salehi E, Zandieh Z, Rashidi M, Taleahmad S, Javedani Masrour M, et al. Comparative evaluation of NOTCH signaling molecules in the endometrium of women with various gynecological diseases during the window of implantation. Iran J Basic Med Sci. 2019;22(4):426–31.

8. Negri L, Lattanzi R, Giannini E, Melchiorri P. Bv8/Prokineticin proteins and their receptors. Life Sci. 2007;81(14):1103–16.

9. Chen B, Yu L, Wang J, Li C, Zhao K, Zhang H. Involvement of Prokineticin 2 and Prokineticin receptor 1 in lipopolysaccharide-induced Testitis in rats. Inflammation. 2016;39(2):534–42.

10. Balasubramanian R, Plummer L, Sidis Y, Pitteloud N, Martin C, Zhou Q, et al. The puzzles of the prokineticin 2 pathway in human reproduction. Mol Cell Endocrinol. 2011;346:44–50.

11. Szatkowski C, Vallet J, Dormishian M, Messaddeq N, Valet P, Boulberdaa M, et al. Prokineticin receptor 1 as a novel suppressor of preadipocyte proliferation and differentiation to control obesity. PLoS One. 2013;8(12):e81175.

12. Qureshi R, Kindo M, Arora H, Boulberdaa M, Steenman M, Nebigil C. Prokineticin receptor-1-dependent paracrine and autocrine pathways control cardiac tcf21 fibroblast progenitor cell transformation into adipocytes and vascular cells. Sci Rep. 2017;7(1):12804.

13. Battersby S, Critchley H, Morgan K, Millar R, Jabbour H. Expression and regulation of the prokineticins (endocrine gland-derived vascular endothelial growth factor and Bv8) and their receptors in the human endometrium across the menstrual cycle. J Clin Endocrinol Metab. 2004;89(5):2463–9.

14. Batista Pedro J, Chang HY. Long noncoding RNAs: cellular address codes in development and disease. Cell. 2013;152(6):1298–307.

15. Chenguang G, Maquat LE. lncRNAs transactivate STAU1-mediated mRNA decay by duplexing with 3′ UTRs via Alu elements. Nature. 2011;470(7333):284–8.

16. Ransohoff JD, Wei Y, Khavari PA. The functions and unique features of long intergenic non-coding RNA. Nat Rev Mol Cell Biol. 2017;19(3):143–57.

17. Ni N, Li Q. TGFβ superfamily signaling and uterine decidualization. Reprod Biol Endocrinol 2017;15(1):84.

18. Meng N, Yang Q, He Y, Gu W, Gu Y, Zhen X, et al. Decreased NDRG1 expression is associated with pregnancy loss in mice and attenuates the in vitro decidualization of endometrial stromal cells. Mol Reprod Dev. 2019;86(9):1210–23.

19. Peter Durairaj R, Aberkane A, Polanski L, Maruyama Y, Baumgarten M, Lucas E, et al. Deregulation of the endometrial stromal cell secretome precedes embryo implantation failure. Mol Hum Reprod. 2017;23(7):478–87.

20. Berkhout R, Lambalk C, Repping S, Hamer G, Mastenbroek S. Premature expression of the decidualization marker prolactin is associated with repeated implantation failure. Gynecol Endocrinol. 2020;36(4):360–4.

21. Boulberdaa M, Urayama K, Nebigil C. Prokineticin receptor 1 (PKR1) signalling in cardiovascular and kidney functions. Cardiovasc Res. 2011;92(2):191–8.

22. Arora H, Boulberdaa M, Qureshi R, Bitirim V, Messadeq N, Dolle P, et al. Prokineticin receptor 1 is required for mesenchymal-epithelial transition in kidney development. FASEB j. 2016;30(8):2733–40.

23. Xiao-Huan L, Wen-Bo D, Yue-Fang L, et al. Non-coding RNA LINC00473 mediates decidualization of human endometrial stromal cells in response to cAMP signaling. Sci Rep. 2016;6:22744.

24. Yanni J, Rui C, Yu T, Ruixue Z, Shouqin L, Guo XY, et al. Progesterone-induced RNA Hand2os1 regulates decidualization in mice uteri. Reproduction. 2020;159(3):303–14.

25. Tian F-J, Xiao-Ying H, Jie W, et al. Elevated Tristetraprolin impairs Trophoblast invasion in women with recurrent miscarriage by destabilization of HOTAIR. Mol Ther Nucleic Acids. 2018;12:600–9.

26. Ji-Cui D, Hongguang D, Aldo C, et al. Matrix metalloproteinases and their specific tissue inhibitors in menstruation. Reproduction. 2002;123:621–31.

27. Bany BM, Harvey MB, Schultz GA. Expression of matrix metalloproteinases 2 and 9 in the mouse uterus during implantation and oil-induced decidualization. J Reprod Fertil. 2000;120(1):125–34.

28. Liu H-Z, Liu G-Y, Pang W-W, et al. LncRNA LUCAT1 promotes proliferation of ovarian cancer cells by regulating miR-199a-5p expression. Eur Rev Med Pharmacol Sci. 2020;24:1682–7.

29. Lina W, Dongrun T, Tong W, et al. ELF1-mediated LUCAT1 promotes choroidal melanoma by modulating RBX1 expression. Cancer Med. 2020;9:2160–70.

30. Lin H, Tianan G, Yangjun W, et al. Hypoxia induced LUCAT1/PTBP1 axis modulates cancer cell viability and chemotherapy response. Mol Cancer. 2020;19:11.

31. Zhao Z, Xing Y, Liu Y, Jing S. Lung cancer-associated transcript 1 facilitates tumorigenesis in laryngeal squamous cell carcinoma through the targeted inhibition of miR-493. Mol Med Rep. 2021;23(1):59.

32. Itoh T, Tanioka M, Matsuda H, Nishimoto H, Yoshioka T, Suzuki R, et al. Experimental metastasis is suppressed in MMP-9-deficient mice. Clin Exp Metastasis. 1999;17(2):177–81.

33. Alexander CM, Hansell EJ, Behrendtsen O, Flannery ML, Kishnani NS, Hawkes SP, et al. Expression and function of matrix metalloproteinases and their inhibitors at the maternal-embryonic boundary during mouse embryo implantation. Development. 1996;122(6):1723–36.

Fig. 1 Expression of PK2, PKR1, PRL, and IGFBP1 in the endometrium of control group and RIF group. **A** PK2 and PKR1 levels were measured by qRT-PCR in control and RIF groups; **B** PRL and IGFBP1 levels were measured by qRT-PCR in two groups; **C,D** Western blotting and densitometric analyses of PK2, PKR1, PRL, IGFBP1 protein levels in two groups; ** *P* < 0.01

Fig. 2 Immunolocalization of PK2 and PKR1 proteins in endometrial tissue of two groups. **A**, **B** Expression and localization of PK2 and PKR1 in control group; **C**, **D** Expression and localization of PK2 and PKR1 in the RIF group. The scale bar at the lower-right part of all sections represents 50 μm

Fig. 3 Effect of PK2 overexpression on the in vitro decidualization of T-HESC. **A** Cell morphology changes of T-HESC in Control group and PK2 overexpression group at 0 h, 24 h, 48 h, 72 h after decidualization in vitro. **B** PRL and IGFBP1 were measured by qRT-PCR in two groups, ** P < 0.01

Fig. 4 **A** Clustering diagram of lncRNA expression profiles between two groups. The differentially expressed mRNA expression levels were clustered using lg (FPKM+) values. Red area represents increased expression levels, and green represents decreased expression levels. The arrow indicates LUCAT1. **B**. RT-qPCR for 5 lncRNAs in two groups. The x-axis indicates the names of the mRNAs, and the y-axis indicates mRNA expression (Relative to control) between RIF and control groups

Fig. 5 Protein-Protein Interaction Networks of differential genes

Fig. 6 RNA and protein expression levels of LUCAT1, RAMP2 - AS1 and MMP9 in endometrium of two groups of patients. **A-C** LUCAT1, RAMP2 - AS1 and MMP9 level were measured by qRT-PCR in two groups. **D,E** Western blot and densitometric analyses of MMP9 protein expression in the two groups. * * * *P* < 0.001

Fig. 7 RNA and protein expression levels of LUCAT1 and MMP9 in two cell models. BD(before decidualization): empty vector + no decidualization, AD: after decidualization, CON(control group): empty vector + decidualization, +(PK2 overexpression group): overexpression of PK2 + decidualization, −(PK2-siRNA group): PK2-siRNA + decidualization. RNA expression of **A**-**B**. LUCAT1 and MMP9 in the four groups. **C**,**D**. MMP9 protein expression in four groups

Table 1 Primer sequences

| Name | Primer | Sequence |
| --- | --- | --- |
| GAPDH | Forward5’-CGCTTCGGCAGCACATATAC −3′  Reverse5’-AAATATGGAACGCTTCACGA − 3′ | |
| PRL | Forward5’-GCCTCTGTATCATCTGGTCACG − 3′  Reverse5’-TGCGTAGGCAGTGGAGCAG − 3′ | |
| IGFBP1 | Forward5’-CTGCGTGCAGGAGTCTGA − 3′  Reverse5’-CCCAAAGGATGG AATGATCC − 3′ | |
| PK2 | Forward5’-TTGGGCGGAGGATGCA − 3′  Reverse5’-AAATGAAGTCCGTAAACAGGCC − 3′ | |
| PKR1 | Forward5’-TCTTACAATGGCGGTAAGTCCA − 3′  Reverse5’-CTCTTCGGTGGCAGGCAT − 3’ | |
| MMP9 | Forward5’-TCAAGTGGCACCACCACAAC-3′  Reverse5’-TGTACACGCGAGTGAAGGTGA-3′ | |
| LUCAT1 | Forward5’-AAGGTCCATATTAAACGTCCTACAA-3′  Reverse5’-TAGCCATTAGACTGCCAGAGGA-3’ | |
| RAMP2-AS1 | Forward5’-CTCTGGAGTCGGGAGAAGGA-3′  Reverse5’-TGGCACATGAAGTCGCACAC-3’ | |
